# How marketing mix (7Ps) affect the patients’ selection of a hospital: experience of a low-income country

**DOI:** 10.1186/s42506-020-00052-z

**Published:** 2020-09-29

**Authors:** Ramin Ravangard, Amir Khodadad, Peivand Bastani

**Affiliations:** 1 Health Human Recourses Research Center, School of Health Management and Medical Informatics, Shiraz University of Medical Sciences, 29 Ghasrodasht Street, Shiraz, Iran; 2 Student Research Committee, Shiraz University of Medical Sciences, Shiraz, Iran

**Keywords:** Marketing, Marketing Mix, Private hospital, Corporate public hospital, Low-income country

## Abstract

**Background:**

Applying marketing mix in the hospitals is necessary for their success. It is also important to optimize the price, developing services, increasing health literacy, and improving financial resources. Experts and patients may have different views about the factors that influence the decision for choosing a hospital. This study was conducted to identify the factors in the marketing mix which influence patients’ selection of hospitals in Shiraz, Iran.

**Methods:**

A cross-sectional study involving patients assigned to six hospitals; three selected private and three corporate public hospitals in Shiraz in southern Iran in 2018 composed the study sample. From the inpatients and outpatients referring to these hospitals, 300 patients were included using a stratified sampling method proportional to size. Their views on the status of the selected hospitals regarding the 7Ps model of the hospital marketing mix (product, people, price, place, promotion, process, and physical environment) were assessed using a 5-point Likert scale. Data were collected by administering a validated researcher-developed questionnaire (CVI = 2.65, *α* = 0.929).

**Results:**

Among 44 components of marketing mix according to 7Ps model, “specialty of health service providers” had the best status (mean (SD) 4.15 ± 0.82) from the patients’ viewpoints. Among the 7Ps, “physical environment” and “people” had better status respectively. In contrast, the studied hospitals had the poorest status in “promotion.” Significant relationship was revealed between the private and corporate public hospitals in terms of price, promotion, and process (*P* < 0.05). Pearson’s correlation revealed a direct relationship between all the components of marketing mix in the hospitals (*P* < 0.001).

**Conclusion:**

The studied hospitals have an appropriate status in physical environment and people mix but poor status in promotion mix. It is therefore necessary for these hospitals to pay more attention to the “promotion mix” irrespective of the related costs. Moreover, as “process mix” had a high significant correlation with the other marketing mix, managers can improve their marketing services through correcting their current processes.

## Introduction

By definition, marketing is part of a business operation which encompasses planning, promotion, pricing, and delivery of customer-oriented products or services [1] and is considered as a mix of components necessary for planning and implementing all marketing operations as a whole [2]. A marketing mix is a business tool empowering managers to stay in the global competitive environment [3].

In most organizations, the marketing mix is known as 4Ps: namely product, price, place, and promotion. In addition, customer engagement, physical environment, time, and processes are also effective factors in service delivery. Accordingly, “service marketing mixes” or 7Ps include the aforementioned (product, price, place, promotion), in addition to persons, physical environment, and process [4].

Like all organizations, hospitals as the providers of medical and treatment services should employ marketing components in order to achieve a successful performance [5]. Evidence suggests that marketing mix is of essence to optimize service prices, expand surgical operations, increase health awareness, change service providers’ attitudes, increase hospital’s financial resources, and reduce the communication gap between providers and users [6]. On the other hand, enhancing patient satisfaction is the main goal of hospitals and a factor promoting medication adherence and improving patients’ health status; this is the direct output of the marketing mix in hospitals [7]. Other investigations have reported positive outputs for those hospitals which adhere to marketing principles and establish their plans and programs based on marketing mix components [2, 8].

Many studies have been conducted on the relationship of marketing mix and selecting a hospital by patients in Iran. For example, a study in Isfahan, Iran (2001), found the most and the least important factors in selecting a private hospital by patients to be staff members (i.e., physicians and paramedics) and physical environment, respectively [9]. Nasiripour et al. [10] also emphasized on “place” and “price” as the most significant marketing components in public hospital selection in Iran. In Ardabil, Iran (2014), a study detected 13 factors effective in selecting a hospital and classified them into three groups of hospital services, social factors, and hospital facilities, with the hospital services being the major factor affecting such a selection decision [11]. In hospitals affiliated with the Iranian Army (2016), the authors claimed that two factors “physicians and staff,” followed by “clinic facilities,” had the greatest impact on patients’ willingness to choose a clinic [12].

In developed countries, the most important factors according to patients’ perspective differ. For example, a study in London, England (2005), listed factors such as high success rate of surgery, high sanitary standards, effective communication between hospitals and physicians, surgeons’ reputation, post-surgery care at home, hospital reputation, short waiting time, feasible visits for friends and family, ease of access to the hospital, short distance from home, free transfer, free comforts for the patient’s companion, and private and teaching hospitals as the most important factors influencing the patients’ selection in turn [13]. In developing countries, a different ranking of factors was reported by patients. For example, a study in Vietnam (2017) declared that factors such as comfort, specialties, reputation, and word of mouth were the factors effecting hospital choice [14].

In Iran, Abedi and Abedini [2] have identified 7 marketing mix that influence choice. Their results showed that in public hospitals, the price, products, physical assets, place, process, people, and promotion were the determining factors. Meanwhile, in private hospitals, products, physical assets, promotion, place, process, people, and price were a higher priority respectively [2].

Considering the variations of factors among different studies, and given that marketing mix can be an important tool in improving the status of costly hospitals, especially in low- and middle-income countries [15], identification of the main factors affecting the hospital service’s marketing is of essence. Furthermore, as there were no previous studies in this field in Shiraz which is the largest referral metropolitan region in southern Iran, this study aimed to identify the factors in the marketing mix that influence patients’ selection of a hospital in selected hospitals of Shiraz, Iran. The results may provide key information that guide the development of optimal marketing strategies for public corporate and private hospitals and thus enhance patient satisfaction and increase hospitals’ share in the competitive market.

## Methods

### Study design and setting

This cross-sectional study was conducted in 2018. Among all the hospitals in Shiraz, six (three private hospitals and three corporate hospitals), which had a lot of marketing activities, were included in the study. It should be noted that these six hospitals were similar to each other in terms of their in(out)patient wards and their sub-specialties.

### Sample

Assuming *N* = 716,992 (as the total number of patients in the above 6 hospitals in the year 2018), *d* = 0.05, *P* = *q* = 0.5, *α* = 0.1, and 10% patient dropout, the sample size was determined as 300 patients. As the number of in(out)patients referred to these six hospitals differed, a stratified sampling method proportional to hospital size was used to determine the number of patients selected from each hospital so that each was considered a stratum based on the number of patients referred to it. Then, in each hospital, the determined sample size was selected from the patients through a simple random sampling method using the patient sampling frame, including all in(out)patients’ characteristics in general, taken from the hospital admission unit and the random numbers table. Verbal informed consent was obtained from all patients participating in this study.

### Data collection

Data was collected via interview using a questionnaire containing two sections. The first included demographic information (patients’ age, gender, level of education, occupation, and income level), while the second comprised 44 items on various marketing indicators (services, place, price, promotion, process, person, and physical environment) of the hospital health services. These were scored based on a 5-point Likert scale (1 = very weak, 2 = weak, 3 = moderate, 4 = good, and 5 = very good). The content validity of the questionnaire was approved by experts (CVI = 2.65), and its reliability was confirmed using Cronbach’s alpha coefficient (*α* = 0.925).

### Statistical analysis

The data were analyzed using descriptive (mean and standard deviation) and inferential statistics (*T* test, Pearson’s correlation coefficient, and linear regression) with SPSS software version 24.

## Results

There were 167 male (55.7%) and 133 female participants in this study (*N* = 300). Approximately half (50.7%) were aged 30–50 years, and the majority (76.0%) had completed undergraduate-level education. Over a third (*n* = 115, 38.3%) of participants had no permanent job. Among the participants, there were 108 people (36%) with an income level below 10,000,000 Rials as a national currency (approximately 100USD per month (1USD = 100,000 Iranian Rials) (Table 1).

**Table 1:** Characteristics of study participants in six selected Shiraz hospitals 2018 (*N* = 300)

Demographic variables	*N*	%
Sex		
Female	133	44.3
Male	167	55.7
Age (years)		
< 20	17	5.7
20 ≤ 30	91	30.3
30 ≤ 50	152	50.7
≥ 50	40	13.3
Level of education		
Diploma and less than diploma (completing high school)	38	12.7
Bachelor degree	228	76.0
Higher than Bachelor	34	11.3
Job		
Unemployed	115	38.3
Non-governmental employment	77	25.7
Governmental employment	108	36
Level of income		
Less than 100USD/month (less than 10,000,000 Rials)	108	36.0
100–200USD/ month (10,000,000–20,000,000 Rials)	52	17.3
> 200USD/ month (more than 20,000,000 Rials)	140	46.7

Of all the 44 effective indicators of hospital services, physicians’ scientific expertise and professional skills or “specialty of health service providers” with the mean (SD) 4.15(± 0.82) gained the highest ranking from participants. Subsequently, there were physicians’ commitment and their appropriate communication with the patient, proper clothing or uniform used by hospital staff, and the availability of medical expertise and medical services required by patients each with a mean score of 4.13 (with SD = 0.778, 0.856, 0.778, respectively). On the other hand, the lowest scores (3.01 ± 1.00 and 3.07 ± 1.22) were achieved for holding meetings to introduce the hospital’s capabilities and free services respectively (Table 2). Table 2 reports the perspective of hospital patients. The physical environment (with the mean score of 3.94 ± 0.65) was the most highly ranked factor of the 7P. The people (mean score of 3.85 ± 0.63) was the second most effective element in hospital services marketing. Among the seven marketing mix components, promotion (mean score of 3.34 ± 0.68) had the lowest score.

**Table 2:** Mean and standard deviation of marketing mix components

Marketing mix	Components	Mean ± SD	Mean ± SD	*Z* score
Product	Sub-specialists of medicine	4.13 ± 0.78	3.49 ± 0.662	+ 0.75
	Quality of services	3.94 ± 0.85		
	Update and new services	3.37 ± 1.05		
	Consulting services	3.30 ± 1.15		
	Appropriate preventive services	3.32 ± 1.10		
	Home care after discharge	3.34 ± 1.10		
	Telemedicine	3.19 ± 1.14		
	Waiting lists for needed services	3.20 ± 1.22		
	Differentiated services of the hospitals	3.67 ± 1.01		
Place	The location of hospital based on the population	3.82 ± 1.05	3.54 ± 0.730	+ 0.75
	Appropriate transportation	3.70 ± 1.07		
	Possibility of delivering portable services	3.12 ± 1.0		
People	Adequate number of people for service delivery	3.81 ± 0.96	3.85 ± 0.628	+ 1.36
	Attention of staff to mental needs of the patients	3.54 ± 1.18		
	Good relationship between staff and patients	3.65 ± 1.11		
	Having the spirit of criticism, sunniness and commitment	3.49 ± 1.15		
	Scientific skills of the staff	3.78 ± 0.94		
	Using uniforms based on the hospital’s uniforms	4.13 ± 0.86		
	Scientific experience and skills of physicians	4.15 ± 0.82		
	Practitioners’ commitments and their relationship with patients	4.13 ± 0.78		
	Useful recommendations to patients by nurses and doctors	3.94 ± 0.85		
Promotion	Good reputation	3.37 ± 1.05	3.34 ± 0.675	+ 0.50
	Using banners and advertising tools	3.30 ± 1.15		
	Using websites	3.32 ± 1.10		
	Using brochures to introduce hospital’s services	3.34 ± 1.10		
	Periodic reports for the success and achievements	3.19 ± 1.14		
	Using TV advertisements and telephone	3.20 ± 1.22		
	Applying ceremonies to introduce hospital’s potentials	3.01 ± 1.0		
Price	Appropriate tariff fees comparing the other centers	3.80 ± 0.97	3.49 ± 0.812	+ 0.60
	Contract with basic and complementary insurances	3.82 ± 1.04		
	Waive for the poor patients	3.20 ± 1.25		
	Transparent and on time bills of the patients	3.56 ± 1.09		
	Free services	3.07 ± 1.22		
Process	Using HIS^#^ and other networks	3.61 ± 0.92	3.59 ± 0.666	+ 0.89
	Attention to patients’ comments and complains	3.16 ± 1.08		
	Describing the details of services to the patients	3.53 ± 10		
	Discipline and carefulness in service delivery	3.82 ± 0.88		
	Speed of service delivery by the staff	3.85 ± 0.96		
Physical environment	Beautifulness and well designing of internal sections	3.90 ± 0.91	3.94 ± 0.652	+ 1.45
	Good air condition system and sufficient light	3.99 ± 0.86		
	Nice external view of the hospital	4.01 ± 0.93		
	Appropriate size of the yard and parking lot	4.02 ± 0.87		
	Appropriate diagnostic and treatment equipment	3.90 ± 0.95		
	Good communication and responding	3.84 ± 1.01		

^#^Hospital Information System

As shown in Table 3, there is a significant difference between the private and corporate public hospitals in terms of price (mean (SD) 3.53 (0.81), *P* = 0.014), promotion (mean (SD) 3.35 (0.69), *P* = 0.009), and process (mean (SD) 3.60 (0.68), *P* = 0.022), with a better status in the corporate public hospitals and provided higher levels of patient ranking.

**Table 3:** The relationship between marketing mix and hospital type (private/corporate hospital), Shiraz, Iran

Marketing mix	Hospital type	Mean ± SD	*P* value*
Product	Corporate	3.50 ± 0.67	0.082
	Private	3.37 ± 0.46	
Physical environment	Corporate	3.96 ± 0.65	0.543
	Private	3.75 ± 0.73	
Price	Corporate	3.53 ± 0.81	0.014*
	Private	2.89 ± 0.49	
People	Corporate	3.86 ± 0.62	0.596
	Private	3.75 ± 0.74	
Promotion	Corporate	3.35 ± 0.69	0.009*
	Private	3.16 ± 0.40	
Place	Corporate	3.54 ± 0.73	0.825
	Private	3.56 ± 0.70	
Process	Corporate	3.60 ± 0.68	0.022*
	Private	3.41 ± 0.39	
Total	Corporate	3.63 ± 0.52	0.123
	Private	3.41 ± 0.35	

*P value according to independent *t* test

Table 4 indicates a significant relationship between the level of education with process (*P* = 0.014), place (*P* = 0.006), and promotion (*P* = 0.008). Moreover, there is a significant relationship between occupation and income level with place (*P* = 0.005 and *P* = 0.033, respectively).

**Table 4:** The relationship between demographic variables and 7Ps

Marketing mix	Mean ± SD	Age (*P* value)	Sex (*P* value)	Education (*P* value)	Employment (*P* value)	Income (*P* value)
Product	3.50 ± 0.662	0.881	0.703	0.085	0.750	0.462
Physical environment	3.94 ± 0.652	0.867	0.675	0.560	0.675	0.623
Price	3.49 ± 0.812	0.586	0.536	0.303	0.688	0.894
People	3.85 ± 0.628	0.914	0.150	0.463	0.250	0.984
Promotion	3.34 ± 0.675	0.877	0.315	0.008*	0.732	0.980
Place	3.54 ± 0.730	0.743	0.739	0.006*	0.005*	0.033*
Process	3.59 ± 0.666	0.426	0.780	0.014*	0.605	0.468

*Significant

Linear regression analysis for the independent variable (patients’ demographic information) and the dependent variable (total score of marketing) in these hospitals showed a strong, inverse relationship (*P* = 0.003 and *β* = − 0.199) between the patients’ level of education and their views to hospital marketing status as patients with higher education levels evaluated the hospital marketing status to be weaker (Table 5).

**Table 5:** Linear regression between patients’ demographic variables and total score of marketing

Model	Unstandardized coefficients		Standardized coefficients	*T*	Sig.
	*B*	Std. error	Beta		
(Constant)	4.132	.191		21.645	0.000
Age	− .058	.040	− .086	− 1.438	0.151
Sex	− .022	.067	− .021	− .324	0.746
Education	− .199	.066	− .190	− 3.025	0.003*
Employment	.086	.049	.144	1.761	0.079
Income	− .047	.044	− .084	− 1.066	0.287

*Significant

Finally, Pearson’s correlation test for different groups of marketing mix components in the selected hospitals revealed a direct relationship between all the concerned elements. The strongest correlation was found between the service and staff (*P* < 0.001), followed by process and promotion (*P* < 0.001), process and physical environment (*P* < 0.001), and process and staff with correlation coefficient of 0.578 (*P* < 0.001). In this regard, the findings suggested that the process, in general, had greater correlation with all other aspects of marketing mix (Table 6).

**Table 6:** Correlation between marketing mix in selected hospitals

	Product	Physical environment	Price	People	Promotion	Place	Process
Product	1	0.435 *P* < 0.001	0.339 *P* < 0.001	0.616 *P* < 0.001	0.561 *P* < 0.001	0.461 *P* < 0.001	0.550 *P* < 0.001
Physical environment		1	0.510 *P* < 0.001	0.538 *P* < 0.001	0.471 *P* < 0.001	0.372 *P* < 0.001	0.579 *P* < 0.001
Price			1	0.372 *P* < 0.001	0.425 *P* < 0.001	0.323 *P* < 0.001	0.471 *P* < 0.001
People				1	0.462 *P* < 0.001	0.426 *P* < 0.001	0.578 *P* < 0.001
Promotion					1	0.398 *P* < 0.001	0.586 *P* < 0.001
Place						1	0.400 *P* < 0.001
Process							1

## Discussion

According to the study findings, the most important component for patients in all the selected hospitals were physical environment, followed by the component “staff” (people), which encouraged patients to select these hospitals. In their study, Jalili et al. [11] in Ardabil, Iran, mentioned that patients assessed the physical environment of rooms and facilities for patients and their companions in the private sector positively. They also assessed the services provided by nursing staff in private hospitals to be highly positive, which is a factor affecting the patients’ willingness to use private hospitals [11]. Yaghoubi et al. [9] in Isfahan, Iran, also identified medical staff (physicians and nurses) as the most important factor in selecting a state hospital by patients, while the physical environment of the hospital was considered as the least important factor among other marketing mix elements. Mwang’s study [16] on the private hospitals in Nairobi also highlighted the staff’s knowledge and experience, their responsiveness to service provision, and staff’s explanation of the patient’s medical conditions as the three main subcomponents of the staff marketing mix component, especially for the hospitals in low-income countries, where little cost could be spent on promotion or physical environment.

Following the elements “staff” and “physical environment,” another marketing mix component which was assessed positively from the patients’ perspective was “process,” one of the most important indicators of which was quick service provision by the medical staff. In this regard, Nasiripour et al. [17] in four private and public hospitals located in Sari, Iran, assessed the private hospitals to be better in terms of providing services in a shorter timeframe and found a meaningful relationship between the speed of service provision and the private services of the hospitals. Hosseini et al. [18] in their study in private hospitals in Tehran, Iran, considered discipline and operation speed in providing services as the second most effective factor in attracting patients in private hospitals, following the physicians and nurses’ constant presence on patient’s bed.

The place was ranked fourth in the selected hospitals. In this regard, hospital’s location indicators as well as adequate transport systems for patients’ easy access to the hospital was also assessed positively by the patients. Rao et al. identified the availability of medical services as one of the main dimensions of the perceived quality for patients [19]. Nasiripour et al. [10] also considered place or access to services as one of the main factors affecting the services marketing.

Another marketing dimension, which was investigated in this study, was service marketing mix components, i.e., “product.” The importance of this marketing mix is considerable because it may lead to patients’ promoted satisfaction [20]. The most significant service delivery indicator, which was highly acknowledged by the participants of the present study was medical expertise and services. Ahmad et al. [21] in their study on private hospitals in Jeddah, Saudi Arabia, considered health care strategy as the first factor affecting patients’ satisfaction with hospital performance. In their research on Lebanese hospitals, Aoun and Alaaraj [20] also demonstrated that, from the perspective of the patients, the services provided in Lebanese hospitals, in particular advanced technology services, along with the use of trained staff and appropriate pricing strategies play a critical role in the marketing of hospitals in developing countries.

The findings of the present study revealed that “price” played a less remarkable role in patient decision of selecting a hospital. Regarding this marketing component, the patients’ evaluation of the health service tariff fees compared to other hospitals and the contract with complementary and basic health insurance companies was high. However, their assessment of the free services and discounts for low-income patients was moderate. This is strongly influenced by government policies to terminate the health insurance contract with private hospitals which require patients to pay for receiving private services. Nasiripour et al. [10] also showed that discounts on patient costs could be effective in healthcare marketing. Lee and Shi in Taiwan also regarded the cost variation strategy effective in healthcare marketing [22]. On the contrary, Ahmad et al. [21] in Saudi Arabia stated that price strategies had no effect on the patient satisfaction. This inconsistency could be due to the high per capita income of individuals in the concerned country.

Finally, promotion had a lower score than the other marketing mix components in the hospitals, implying that the hospitals have acted inadequately in this regard. The hospitals should take more advantage of appropriate promotion tools in order to promote and develop the hospital services and facilities. Azimi et al. [23] in their study in Iran considered promotional activities a critical factor in marketing and attracting medical tourism. However, Nitin et al. [24] found that the tertiary-level hospitals in India were less eager to use marketing mix components, including promotion.

The other finding of this study was the positive correlation between the marketing mix elements. Of note, “process” had the greatest correlation with other marketing mix elements. Ahmad et al. [21] also reported a positive correlation between different marketing strategies. Moreover, there was a strong, inverse relationship between the patients’ level of education and their viewpoint toward hospital marketing status as patients with higher education levels gave lower scores to the evaluation of the hospital marketing status compared to patients with lower levels of education. Possible reasons for this are varied but may be due to the individuals’ increased awareness of various marketing tools, services in other communities, new medical technologies, and global standards in the field of health services, facilities, and hoteling options of other hospitals in other countries. Yaghoubi et al. [9] also found a relationship between patients’ level of education and various marketing mix components such as price, place, and promotion.

In sum, it seems that people and physical environment have the highest status among marketing mix in the present study. However, the significant differences were seen among price, promotion, and process between private and public corporate hospitals (*P* < 0.05). In this regard, Yaghoubian et al. [25] showed that price is the key to receiving health services and that low price and an appropriate place are important in increasing patients’ attitude to using different primary services provided by hospitals and health-care centers especially in developing countries.

### Limitations

One of the limitations in this study is the inconsistency in the approach followed to collect the data as data collectors opted sometimes to explain some questions to the patients with low level of education. This may have resulted in some variation in the participants’ answers to the questions. Another limitation is not considering other factors that may affect the patient’s decision to select the hospital such as the diagnosis and the urgency of care needed.

## Conclusion

The “promotion” component of the marketing mix was the least influential factor on the decision of patients for selecting the hospital while “physical environment” was the most important factor affecting selection. The “specialty and skills of health providers” was also one of the main factors affecting patients’ preferences to choose the hospitals. According to these results, the senior managers of these hospitals are recommended to keep the current status in terms of physical environment and people and to adopt some measures to further motivate their employees. Regarding the prices, they need to adopt appropriate strategies to provide greater satisfaction of service recipients and community members. Relevantly, they could enhance their patients’ satisfaction with the establishment of different centers for preventive services, health education, counseling, and some free health care services as well as competitive prices. These hospitals should also have optimal promotion strategies such as television advertising, banners, brochures, and hospital website to advertise the hospital equipment, facilities, expertise, and services for patients and society.

## Data Availability

Data are available on reasonable request.
